# Development of a biomarker database toward performing disease classification and finding disease interrelations

**DOI:** 10.1093/database/baab011

**Published:** 2021-03-11

**Authors:** Shaikh Farhad Hossain, Ming Huang, Naoaki Ono, Aki Morita, Shigehiko Kanaya, Md Altaf-Ul-Amin

**Affiliations:** Computational Systems Biology Lab, Graduate School of Science and Technology, Nara Institute of Science and Technology (NAIST), 8916-5, Takayama, Ikoma, Nara 630-0192, Japan; Computational Systems Biology Lab, Graduate School of Science and Technology, Nara Institute of Science and Technology (NAIST), 8916-5, Takayama, Ikoma, Nara 630-0192, Japan; Computational Systems Biology Lab, Graduate School of Science and Technology, Nara Institute of Science and Technology (NAIST), 8916-5, Takayama, Ikoma, Nara 630-0192, Japan; Computational Systems Biology Lab, Graduate School of Science and Technology, Nara Institute of Science and Technology (NAIST), 8916-5, Takayama, Ikoma, Nara 630-0192, Japan; Computational Systems Biology Lab, Graduate School of Science and Technology, Nara Institute of Science and Technology (NAIST), 8916-5, Takayama, Ikoma, Nara 630-0192, Japan; Computational Systems Biology Lab, Graduate School of Science and Technology, Nara Institute of Science and Technology (NAIST), 8916-5, Takayama, Ikoma, Nara 630-0192, Japan

## Abstract

A biomarker is a measurable indicator of a disease or abnormal state of a body that plays an important role in disease diagnosis, prognosis and treatment. The biomarker has become a significant topic due to its versatile usage in the medical field and in rapid detection of the presence or severity of some diseases. The volume of biomarker data is rapidly increasing and the identified data are scattered. To provide comprehensive information, the explosively growing data need to be recorded in a single platform. There is no open-source freely available comprehensive online biomarker database. To fulfill this purpose, we have developed a human biomarker database as part of the KNApSAcK family databases which contain a vast quantity of information on the relationships between biomarkers and diseases. We have classified the diseases into 18 disease classes, mostly according to the National Center for Biotechnology Information definitions. Apart from this database development, we also have performed disease classification by separately using protein and metabolite biomarkers based on the network clustering algorithm DPClusO and hierarchical clustering. Finally, we reached a conclusion about the relationships among the disease classes. The human biomarker database can be accessed online and the inter-disease relationships may be helpful in understanding the molecular mechanisms of diseases. To our knowledge, this is one of the first approaches to classify diseases based on biomarkers.

**Database URL:**  http://www.knapsackfamily.com/Biomarker/top.php

## Introduction

**Figure 1.**
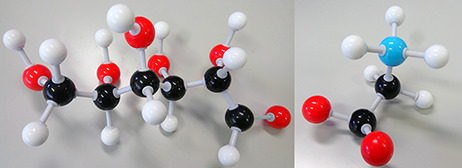
Disease biomarkers; Black, Grey, Red and Blue sphere colors correspond to C, H, O and N, respectively. (i) Diabetes biomarker (Glucose—C_6_H_12_O_6_). (ii) Encephalopathy biomarker (Glycine—C_2_H_5_NO_2_).

A biomarker (short for biological marker) (1) is defined as a biochemical, cellular, gene related or molecular alteration that is measurable (2) in biological media, such as blood, body fluids, tissues or cells by which diseases can be identified. Figure 1 shows two examples of biomarkers.

A biomarker is an indicator of a disease or disease symptom, which indicates the normal or abnormal condition of a body. Previously, markers of prognosis were considered as biomarkers. Now the concept of biomarker has become widespread. Clinical test results, behavioral or cognitive functioning test results, growth or other physical measurements are also considered biomarkers (3). In exposure studies, the use of biomarkers is referred to as biomonitoring. Biomarkers are generally classified into three categories, which are exposure, effect and susceptibility. Biomarkers of exposure involve concentrations of the susceptibility characteristics, actual chemicals or chemical metabolites, chemical residues, exogenous parent chemical, DNA, protein or changes in the body fluids or tissues (4, 5). Biomarkers of effect are the quantifiable changes, which show an exposure to a compound and may show a resulting health effect (6). Biomarkers of susceptibility indicate the detection of a polymorphism or particular genotype or a natural characteristic of an organism (7).

The usage of biomarkers is increasing in many health areas such as diagnosing, clinical practice, monitoring disease, ingredient prediction for novel drugs and precision medicine (PM). In clinical development, biomarker assays are becoming more important and are used to understand the mechanism of action of a drug as a surrogate marker for monitoring clinical efficacy. It has significant importance in PM and is helpful to treat adverse drug reactions (8). According to the PM coalition, there were 132 personalized medicines in the market in 2016, compared with just five in 2008 and 27% of the new molecular entities approved by the FDA in 2016 can be classified as a PM (https://invivo.pharmaintelligence.informa.com/IV005059/Personalized-Medicine-An-Infographic).

Disease patterns change constantly, and identification of accurate biomarkers is also an important challenge. For finding and predicting active medicines, researchers need to read the case studies, mining big data from scattered documents. It is tough to find the right drug, for the right patient, within the right time period. Recently, data management of biomarkers has become a crucial topic because biomarkers are playing significant roles in various disciplines of health research. In this situation, a good quality biomarker database may be a potential solution for the challenge (9). In medical data science biomarker data are going to get much bigger because of the rapid increase of large-scale omics information produced by metabolomics, proteomics, etc. With the growing data volume, the development of a biomarkers database has become a very important issue in the health care field as presently biomarkers are used to detect various human diseases. It is a demand of the time to have an easy-access single platform where biomarker data will be stored that will provide more accurate and large-scale information as a time-saving tool for drug research. However, some biomarkers databases can be found on the web those are not comprehensive or free (e.g. GOBIOM (https://www.gobiomdbplus.com/about-us), BioAgilytix (https://www.bioagilytix.com/biomarker-menu/), Charles River (https://wwwapps.criver.com/BiomarkersDB/) and upbd (http://upbd.bmicc.cn/biomarker/web/indexdb)).

## The KNApSAcK biomarker database

**Figure 2.**
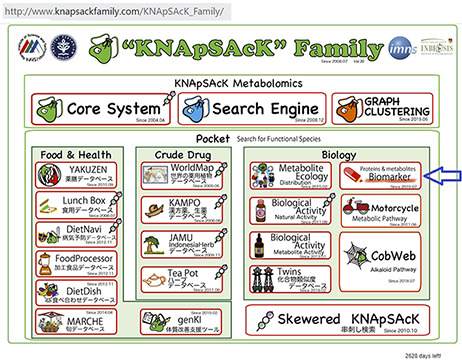
The main window of the KNApSAcK family databases and the arrow indicating the biomarker icon.

We have accumulated 4539 disease-biomarker associations involving 2181 biomarkers and developed the KNApSAcK human biomarker database. This database is linked with the KNApSAcK Core (Figure 2) and the KNApSAcK Metabolite Activity Database (10, 11).

Unambiguous and credible biomarkers data were collected from various reliable sources such as the National Center for Biotechnology Information (NCBI), published patents, proceedings of conferences, approved documents, Google Scholar and other recognized documents. The references were hyperlinked to ensure the reliability of the data.

Biomarkers and references were primarily selected according to the following criteria:

1. Biomarker definitions by the National Institutes of Health (NIH) were followed (1).
2. PubMed, Scientific Conferences and regulatory-approved documents were only considered as the biomarker data source.
3. After the initial selection, the articles were examined by our group.
4. Mainly exposure types of biomarkers were considered.

**Figure 3.**
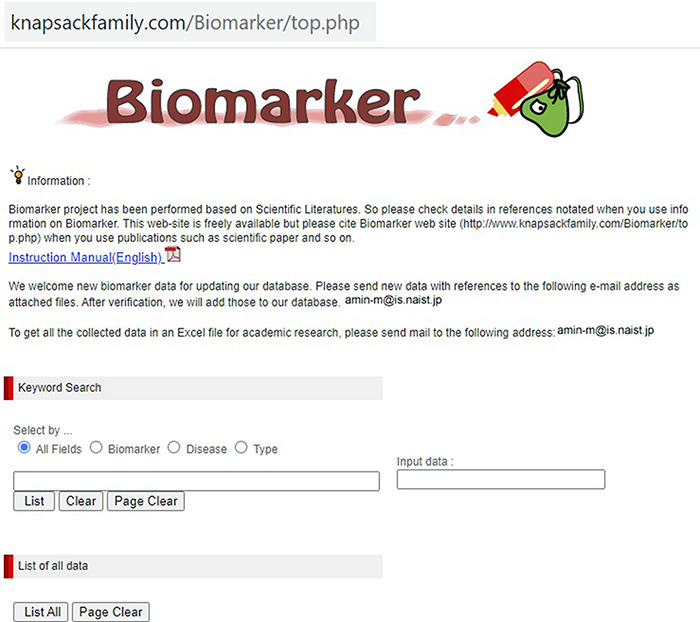
The main window of the biomarker database.

**Figure 4.**
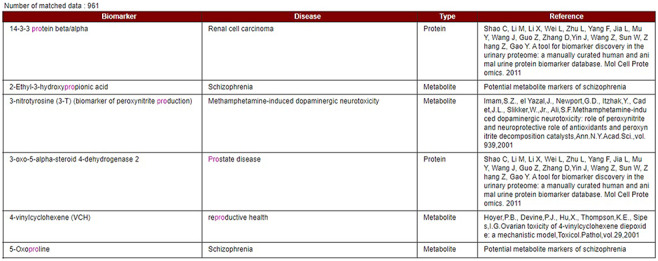
Data display based on partial or exact string matching search.

Our developed human biomarker database is a very rich and up-to-date database where users or researchers can get detailed information about human biomarker data in a single platform. To keep the database updated, we have added an e-mail option in our database page where information on novel biomarkers can be e-mailed. After verification by our team, novel biomarkers will be added to the database by admin. As shown in Figure 2, the database can be accessed by browsing to the page of the KNApSAcK family database (http://www.knapsackfamily.com/KNApSAcK_Family/) and then by clicking the ‘biomarker’ button indicated by an arrow. Figure 3 shows the main page of the biomarker database. Alternatively, the database can be accessed by browsing to the direct database webpage link http://www.knapsackfamily.com/Biomarker/top.php. The database has two types of search options called (a) keyword-based data search and (b) all data search. As shown in Figure 3, for the keyword-based data search, clicking a radio button can select one of the four options [(i) all fields, (ii) biomarker, (iii) disease and (iv) type]. For example, as partially shown in Figure 4, after entering the term ‘pro’ and selecting the radio button ‘all fields’, if the ‘list’ button is clicked, a table appears. The database retrieves data based on exact or partial string matching. More detailed descriptions of the search options are available in the instruction manual, which can be downloaded by clicking on the indicated location on the online page of the database (Figure 3). Features of our Biomarker Database are as follows:

1. Quick and easy access
2. Online data view without registration
3. Interface with comprehensive search features
4. String searching and intelligent analysis
5. Data sharing with no restriction
6. Dedicated server and routine updates

After completion of the database, we have used the disease–biomarker relations for the purpose of disease classification and organized the data for clustering to find disease–disease relations.

**Figure 5.**
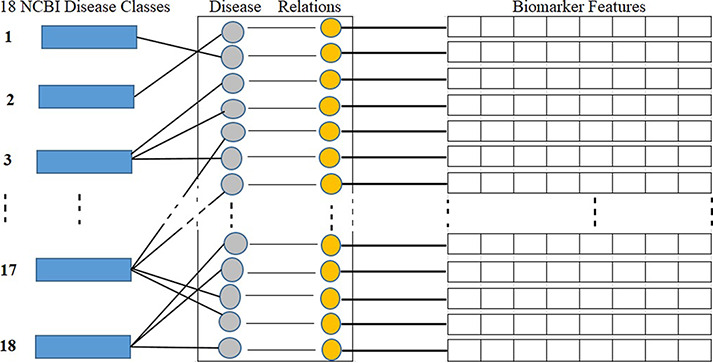
Disease classes, disease-biomarker relations and biomarker feature connectivity.

NCBI is a branch of the NIH of the USA. NCBI defines and classifies diseases into 16 main classes according to symptoms and disease pattern (https://www.ncbi.nlm.nih.gov/books/NBK22183/). In this study, as shown in Table 1, we considered total 18 disease classes in total, where disease classes N1 to N16 are adopted from the NCBI, and N17 and N18 are determined according to a reference paper (12) and represented by the asterisks symbol in the ‘Ref.’ column. As shown in Figure 5, each biomarker and disease relation is studied and mapped into these 18 disease classes as ‘one to many’ relations. As illustrated in Figure 5, biomarkers are also represented by their structural features. Table 1 shows the number, name and collected disease–biomarker relations in the context of the 18 disease classes. The data contain mainly two types of biomarkers as follows: (i) protein biomarkers and (ii) chemical or metabolite biomarkers.

**Table 1.** 18 disease classes and the number of disease–biomarker relations

ID	Ref	Name of disease class	Disease–biomarker relations		Total relations
			Protein	Metabolite	
N1	NCBI	Blood and lymph diseases	151	73	224
N2	NCBI	Cancer	517	338	855
N3	NCBI	The digestive system	82	20	102
N4	NCBI	Ear, nose and throat	90	14	104
N5	NCBI	Diseases of the eye	4	10	14
N6	NCBI	Female-specific diseases	172	68	240
N7	NCBI	Glands and hormones	206	77	283
N8	NCBI	The heart and blood vessels	80	59	139
N9	NCBI	Diseases of the immune system	262	198	460
N10	NCBI	Male-specific diseases	8	7	15
N11	NCBI	Muscle and bone	40	35	75
N12	NCBI	Neonatal diseases	88	31	89
N13	NCBI	The nervous system	71	40	111
N14	NCBI	Nutritional and metabolic diseases	86	68	154
N15	NCBI	Respiratory diseases	268	171	439
N16	NCBI	Skin and connective tissue	57	61	118
N17	*	The urinary system	571	165	736
N18	*	Mental and behavioral disorders	154	197	351

(asterisks refer to reference paper).

Next, biomarker format files were downloaded from NCBI and similarities between biomarkers were calculated based on the biomarker features. A network was constructed by taking similar biomarker pairs, and a graph clustering algorithm was used to determine the clusters in the network. Subsequently, we utilized the clusters as characteristic features for disease classes and applied hierarchical clustering to disease classes considering protein and metabolite biomarkers separately (discussed in section 3). We then compared the dendrograms using Baker’s gamma correlation which is discussed in detail in section 4. Finally, we found significant inter-disease relations among the disease classes that are discussed in section 4.

## Materials and methods

### Classification of disease classes based on biomarkers

Classification enables us to partition a vast expanse of entities into meaningful groups that is otherwise disordered (13). Disease classification can lead to understanding disease mechanisms, developing drugs, choosing medicines and guiding medical practice. Disease classification is an old framework which has continued from the 17th century until now based on different disease criteria and technology advances (14, 15). Taxonomy is fundamental in biology and originated in the 17th century, which uses classification by similar characteristics of individual descriptions among the animal world (16–18). Sydenham notes 1685 disease symptoms and established a hierarchy in which diseases, symptoms and the related botany of the treating herbs are linked (https://archive.org/details/b24400750). In the 18th century, de Sauvages clustered diseases by emphasizing a patient symptom-centric structure (19). In the nineteenth century, laboratory information, clinical signs, radiographs and electrocardiography were added to recognize disease type and classification. Bertillon recorded the ‘cause of death,’ especially for infection-related deaths and diseases were classified based on the organ system (20). In more recent times, the use of high computation facilities and big data involving mRNAs, genes, and metabolites are the basis for classifying diseases, for understanding interactions among diseases and for prediction of drug ingredients (21–23).

In the present work, we are classifying diseases by an upper hierarchy, i.e. based on 18 disease classes. This upper level classification is good for less noisy interpretations of disease relations and avoiding overfitting. Also, Table 1 implies that different disease classes are associated with different numbers of biomarkers, i.e. some are linked to many biomarkers whereas others are linked to a small number of biomarkers. Furthermore, the biomarker data we collected are not comprehensive and many new biomarkers will be found in future. Therefore, to compensate for the incompleteness and imbalance of the data, we determined structurally similar clusters of biomarkers and utilized those clusters as features of the disease classes.

The 18 disease classes were classified twice, once based on protein biomarkers and then again based on metabolite biomarkers. We have adopted two similar procedures separately which are explained in the following sections, and finally, the results are compared based on Baker’s gamma correlation.

### Classification of disease classes based on protein biomarkers

A sequence similarity in proteins indicates a functional similarity to a certain extent (24). A similarity in sequences increases the likelihood of proteins being involved in similar or related signaling and metabolic pathways (25). Therefore, classification of diseases based on protein biomarkers will obviously be helpful to provide insight into disease mechanisms at the molecular level. When mechanisms are known, it leads to narrowing down potential drug candidates for a disease.

To find disease classifications and inter-disease relations, protein biomarker data are formatted, mapped to disease classes, protein descriptors are extracted, clustered and a disease versus clusters matrix is formed. The six adopted steps are discussed below.

#### Formatting data concerning the protein biomarkers

As indicated in Table 2, the protein biomarkers, respective diseases and references are arranged in a tabular format (as indicated in Table 2). In our data, the number of unique protein biomarkers is 1686 and the protein–disease associations are 3693, because one protein may have associations with multiple diseases.

**Table 2.** Protein biomarkers, accession ID, related diseases and references

Serial No.	Protein biomarker	Accession ID	Disease name	References
1	Alpha 1-fetoprotein (AFP)	P02773.1	Hepatic cancer	Tatekawa,Y., Asonuma,K., Uemoto,S., Inomata,Y., Tanaka,K. Liver transplantation for biliary atresia associated with malignant hepatic tumors. J. Pediatr. Surg., vol. 36, 2001
2	Alpha-2 haptoglobin	AAA88080.1	Schizophrenia	Rohlff,C. Proteomics in neuropsychiatric disorders. Int. J. Neuropsychopharmacol., vol. 4, 2001
–	–	–	–	–
3693	Caspase-3	NP_990056.1	Gastric cancer	Chen H, Yang X, Feng Z, Tang R, Ren F, Wei K, Chen G. Prognostic value of Caspase-3 expression in cancers of digestive tract: a meta-analysis and systematic review. Int. J. Clin. Exp. Med. 2015;8:10225–10234

The protein biomarkers and disease relations are then classified into the 18 disease classes (mentioned in Table 1). As shown in Table 3, we created a 3693 × 18 matrix where rows represent biomarker–disease relations and columns represent 18 disease classes and we put 1 in a cell if the corresponding disease belongs to the corresponding disease class (N1, N2,…, N18).

**Table 3.** Protein biomarkers and mapping to the 18 disease classes

Serial No.	Protein biomarker	Disease name	N1	N2	–	N18
1	Alpha 1-fetoprotein (AFP)	Hepatic cancer		1		
2	Alpha-2 haptoglobin	Schizophrenia				1
–	–	–	–	–	–	–
3693	Caspase-3	Gastric cancer		1		

#### Recording the accession ID and FASTA file download

The Accession ID (Identification Number) is the unique ID of a Fasta file which contains the linear sequence of amino acids within a protein. For our 1686 protein biomarkers, we collected the Accession IDs from the NCBI URL by manual searching (https://www.NCBI.nlm.nih.gov/protein/). Before recording the Accession IDs, the protein biomarker names in our data and the NCBI names were checked carefully for exact matches. Using the Accession IDs, the FASTA files corresponding to protein biomarkers are downloaded by using NetBeans IDE 8.2 and the JAVA programming language. FASTA files are stored in a searchable descriptor database as a list object. In biochemistry and bioinformatics, a FASTA file corresponding to a protein is a text-based format for representing amino acid (protein) sequences, in which amino acids are represented using single-letter codes. The FASTA format is easy to manipulate and parse the sequences using text-processing tools such as the R programming language, Python, Perl and Ruby. The linear sequence of amino acids is called the primary structure of a protein. Proteins are made of versatile sequences of 20 types of natural amino acids. To represent the 20 amino acids named alanine (A), arginine (R), asparagine (N), aspartic acid (D), cysteine (C), glutamic acid (E), glutamine (Q), glycine (G), histidine (H), isoleucine (I), leucine (L), lysine (K), methionine (M), phenylalanine (F), proline (P), serine (S), threonine (T), tryptophan (W), tyrosine (Y) and valine (V), three-letter codes or single letter codes are used (https://www.ajinomoto.com/aboutus/amino_acids/20-amino-acids). FASTA files are sequences of these 20 amino acids’ single letter codes. The ‘protcheck(x)’ function in the ‘protr’ package is used to check the authenticity of FASTA files, and 30 FASTA files were deleted from the protein biomarker list before generating the descriptors.

#### Dipeptide composition extraction using the ‘protr package’ in R

In the R language, the protr package (https://cran.r-project.org/web/packages/protr/vignettes/protr.html) (26) is a unique and comprehensive toolkit which is used for generating various numerical representation schemes of protein sequences. It is extensively utilized in chemogenomics and bioinformatics research. Amino acid composition, conjoint raid, autocorrelation, quasi-sequence order, composition, transition and distribution, profile-based descriptors derived by position-specific scoring matrix and pseudo-amino acid composition are all included in protr as a common used descriptors list. The protein sequence descriptors function named extractX() is used for amino acid composition descriptor in the protr package where X stands for a descriptor name. There are three amino acid composition descriptors in the protr package as follows: (i) amino acid composition, (ii) dipeptide composition and (iii) tripeptide composition. We examined all three types of compositions for the protein biomarkers dataset to choose the best one for this study. We have found that dipeptide composition is a better descriptor than the other two descriptors. Also, many other studies previously utilized dipeptide compositions to measure the structural similarity between proteins (27–29). Amino acid composition gives the percentages of individual amino acids within the protein that does not contain any information related to sequence pattern and tripeptide composition descriptor results in zero for most attributes. Finally, dipeptide composition descriptors for the 1656 protein biomarkers as a 400-dimensional matrix (Table 4) were calculated by using the function named extractDC(). This is defined as follows:

$$\begin{equation*}f\left( {r,s} \right) = {{{N_{rs}}} \over {N - 1}}\quad r,s = 1,2,3, \ldots ,20\end{equation*}$$

**Table 4.** 400-dimensional descriptors of protein biomarkers

Protein accession ID	AA	RA	NA	–	VV
1AT3_B.fasta	0.020325	0.012195	0	–	0.004065
1BVK_D.fasta	0	0.009346	0	–	0
–	–	–	–	–	–
1E6O_L.fasta	0.014218	0.004739	0	–	0.004739

where *Nrs* is the number of dipeptides represented by amino acid type ‘*r*’ and type ‘*s*’ and *N* is the length of the sequence.

#### Protein biomarker similarity calculation using PCC

**Figure 6.**
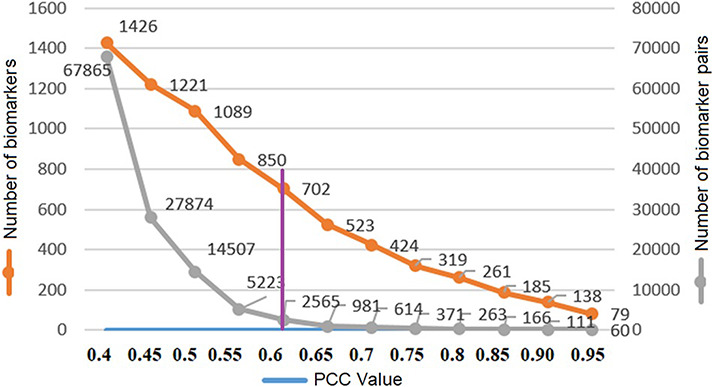
Threshold PCC value selection.

For calculating the structure-based similarity (30, 31) between protein biomarkers, we utilized the Pearson correlation coefficient (PCC) based on 400 dimensional descriptors. PCC was calculated using the following equation:

$$\begin{equation*}corr\left( {X,Y} \right) = {{\mathop \sum \nolimits_{i = 1}^l \left( {Xi - \bar X} \right)\left( {Yi - \bar Y} \right)} \over {\sqrt {\mathop \sum \nolimits_{i = 1}^l {{\left( {Xi - \bar X} \right)}^2}\mathop \sum \nolimits_{i = 1}^l {{\left( {Yi - \bar Y} \right)}^2}} }}\end{equation*}$$

where *X* and *Y* are protein accession IDs and *Xi*, *Yi* are the weights of the *i*th descriptor; $ \bar X$, $\bar Y$ are the corresponding means and $l$ is the descriptor size. The PCC similarity ranges between +1 and −1, where 1 is positive linear correlation, 0 is no linear correlation and −1 is negative linear correlation. The number of the protein biomarker *P* = 1656 so the total number of similarity pairs are (*P*(*P*–1)/2) = (1656(1656–1)/2) = 1 370 340. We are interested in highly positive correlations and therefore, to reduce the computation time, protein pairs with correlation values above 0.4 (67 865 pairs) are saved in a file using the R programming language. Protein biomarker pairs are sorted in descending order, and next, count of biomarkers and protein pairs are plotted with respect to PCC to find the optimum PCC value for this study. In Figure 6, the number of unique protein biomarkers (1426 to 79) and the number of pairs (67 865 to 60) are plotted against the PCC values (0.40–0.95) where the numbers along the left vertical axis indicate protein biomarkers and those along the right vertical axis indicate the number of pairs. We observe that at 0.6 the slope of the curve showing protein pairs is very low. Moreover, based on other studies, the PCC value 0.6 can be considered as a reasonably good correlation similarity (12, 32). Therefore, empirically, we selected 0.6 as the PCC threshold in this study. The number of protein pairs having PCC >0.6 is 2565 which contains 702 unique protein biomarkers (42% of total).

#### Network visualization by Cytoscape and clustering by DPClusO

**Figure 7.**
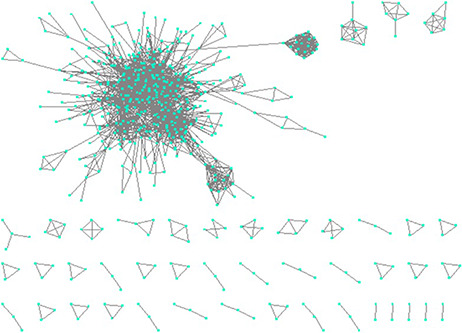
Constructing network based on structural similarity between biomarkers; Nodes represent proteins and edges represent structure similarity.

We visualized the structural similarity-based network of protein biomarkers using Cytoscape (33). Figure 7 shows the network, consisting of 2565 protein biomarker pairs having PCC >0.6. In the network shown in Figure 7, a node is a protein biomarker and an edge represents structural similarity in terms of PCC.

The constructed protein network was clustered by using the graph clustering algorithm DPclusO. DPClusO is a graph clustering algorithm that is used for extracting densely connected nodes as a cluster from a network (34–36). The DPClusO algorithm was developed for the detection of protein complexes in large interaction networks. DPClusO can be applied to an undirected simple graph *G* = (*N, E*) that has a finite set of nodes *N* and a finite set of edges *E*. Density and cluster property are two important parameters in this algorithm. Density *d* is a real number ranging from 0 to 1 and cluster property *cp* of any node *n* with respect to a cluster *k* of density *d_k_* and size *N_k_* is defined as below:

$$\begin{equation*}c{p_{nk}} = {{\left| {{E_{nk}}} \right|} \over {|{d_k}\left| { \times |{N_k}} \right|}}\end{equation*}$$

We applied DPClusO with the following settings: cluster property *cp* = 0.5, density *d* = 0.5 and overlapping coefficient OV = 0.05. DPclusO generated 242 protein clusters.

#### Disease classes versus protein clusters matrix

For the purpose of classifying diseases, we utilize the structurally similar clusters of protein biomarkers as features. One biomarker may belong to multiple clusters because we have applied the DPclusO algorithm which generates overlapping clusters and one biomarker may be associated with multiple diseases. We have made a matrix where rows represent the 18 disease classes and columns represent clusters (Table 5). An element of the matrix is the number of common protein biomarkers associated with the corresponding disease class and the corresponding cluster. The dimensions of this matrix are 18 × 242. This matrix is used to classify disease classes and the classification dendrogram is shown and discussed in section 4.

**Table 5.** 18 disease classes versus protein cluster data matrix

	P_Cluster1	P_Cluster2	P_Cluster3	–	P_Cluster242
N1	7	1	4	–	1
N2	41	19	24	–	1
N3	3	4	2	–	1
N4	1	0	1	–	0
–	–	–	–	–	
N17	34	15	10	–	0
N18	10	5	3	–	0

### Classification of diseases based on metabolite biomarkers

Structural similarity in metabolites often results in activity similarity (37, 38). Structurally similar metabolites might be involved in the same or related metabolic pathways. Structurally similar metabolites might be produced by diseases caused by disruptions in similar pathways. Therefore, it is worthwhile to classify diseases based on metabolite biomarkers for revealing molecular level mechanisms and causes behind diseases.

#### Dataset formatting concerning metabolite biomarkers

Metabolite biomarkers, associated diseases and references are arranged in a tabular format (similar to Table 2). In our dataset, the number of unique metabolite biomarkers is 495 and disease-biomarker associations are 846 because one metabolite may be associated with multiple diseases. Disease and biomarker relations are classified into 18 disease classes. The metabolite biomarker dataset is made into an 846 × 18 table where rows are the metabolite biomarker–disease relations and columns are the 18 disease classes. We have put 1 in the cell to indicate an association between the corresponding biomarker and the disease class (similar to Table 3).

#### Atom pairs fingerprint generation for metabolite biomarkers

PubChem IDs (a public repository for information on chemical substances and their biological activities) of metabolite biomarkers are recorded in the dataset and downloaded from https://pubchem.NCBI.nlm.nih.gov/ URL by using NetBeans IDE 8.2 and the JAVA programming language. InChI Key, molecular formula and molecular weight of metabolite biomarkers are also recorded as additional data in the dataset from the NCBI URL by manual searching. Before recording the PubChem ID and associated metadata, collected biomarker names and NCBI biomarker names are checked carefully for exact matches. SDF files are stored in a searchable descriptor database as a list object. SDF provides 2D coordinates for each unique compound structure.

We have used the ChemmineR (v2.26.0) package (https://www.bioconductor.org/packages/release/bioc/vignettes/ChemmineR/inst/doc/ChemmineR.html) (39) to generate atom pair fingerprints from molecular structure description files for the 495 metabolite biomarkers. An atom pair fingerprint is defined by the shortest paths among the non-hydrogen atoms in a molecule. Each path is described by the length of their shortest bond path, the types of atoms in a pair, the non-hydrogen atoms bonded to them and the number of their pi electrons. There are many molecular fingerprints that are used to represent chemical compounds. Commonly used molecular fingerprints are atom pairs (AP, 1024 bits), PubChem (PubChem, 881 bits), CDK (CDK, 1024 bits), Extended CDK (Extended, 1024 bits), Klekota-Roth (KR, 4860 bits), MACCS (MACCS, 166 bits), Estate (Estate, 79 bits) and Substructure (Sub, 307 bits). In this study, we have used atom pairs fingerprints. By calling the PubChem Compound Identifier (CID) as a list and using the functions ‘sdf2ap’ and ‘desc2fp’ with default parameters of ChemmineR, downloaded SDF files are used to generate 1024 bits’ atom pair fingerprints (AP, 1024 bits). Atom pairs fingerprints are binary vectors composed of ‘0’ and ‘1’.

There are some biomarkers in our list that are not actually compounds. These biomarkers are mainly atoms or ions. These biomarkers show all ‘0’ fingerprints because of no bonding with other atoms the ‘Sum’ function in Excel is used to check all 0 cell fingerprints and 63 biomarkers are deleted for the subsequent analysis.

#### Network of metabolite biomarkers and clustering

The Tanimoto coefficient is utilized for calculating the structure-based similarity (40) between metabolite biomarkers based on 1024-bit atom pair fingerprints. The Tanimoto similarity coefficient ranges between the interval 0 and +1. The number of metabolite biomarkers *M* = 432, so the total number of pairs are (*M*(*M*–1)/2) = (432(432–1)/2) = 93 096. The Tanimoto similarity between two compounds is calculated by the following equation:

$$\begin{equation*}Tanimot{o_{A,B}} = {{AB} \over {A + B - AB}}\end{equation*}$$

A and B are the number of features that are related to individual compounds, and AB is the number of features (or on-bits in the binary fingerprint) common in both compounds. For this study, we have selected the threshold Tanimoto coefficient as 0.85 because metabolite compounds having a Tanimoto coefficient >0.85 represent high similarity. Willett (2014) concluded that the Tanimoto coefficient is standard for similarity searching of 2D fingerprints for different molecular structural similarity measurements and also reported that a Tanimoto co-efficient above 0.85 is a good threshold to represent a similar structure (41). We selected 257 metabolite pairs having a Tanimoto similarity more than or equal to 0.85 which contain 30% of the metabolite biomarkers.

In a previous section, we have discussed DPClusO and its default parameter setting. By using the graph clustering algorithm DPClusO, 257 metabolite biomarker pairs are converted into a network where a node is a metabolite biomarker and the edge represents the Tanimoto coefficient similarity. By keeping the same parameter settings, the network is clustered and DPClusO generates 43 clusters.

#### Disease classes versus metabolite clusters matrix

We utilize the structurally similar metabolite biomarker clusters as features for classifying the disease classes. In each cluster, related biomarkers of each disease class are counted and recorded. We have made a matrix where rows represent disease classes and columns represent clusters (Table 6). An element of the matrix is the number of common metabolite biomarkers associated with the corresponding disease class and the corresponding cluster.

**Table 6.** 18 disease classes versus metabolite cluster data matrix

	M_Cluster1	M_Cluster2	M_Cluster3	–	M_Cluster43
N1	3	4	3	–	0
N2	28	11	8	–	0
–	–	–	–	–	–
N17	16	8	6	–	0
N18	7	9	7	–	2

This is an 18 × 43 matrix where columns are related to 43 clusters, and rows are related to 18 disease classes. The format of the matrix is shown in Table 6.

## Results and discussions

In this section, we discuss the hierarchical clustering of disease classes, comparison of dendrograms and relationships of the 18 disease classes found in our study.

### Hierarchical clustering of 18 disease classes

We applied hierarchical clustering for classifying diseases utilizing the disease classes versus biomarker clusters matrices (Tables 5 and 6). We have chosen the hierarchical clustering because it is easy to understand, easy to explain, easy to visualize using dendrograms and enables distance calculation for better interpretation. For hierarchical clustering, we utilized Euclidean distance measure given by the following equation:

$$\begin{equation*}d\left( {i,j} \right) = \sqrt {\mathop \sum \limits_{k = 1}^n {{({M_{ik}} - {M_{jk}})}^2}} \end{equation*}$$

Here, *d*(*i*, *j*), is the distance between *i*th and *j*th disease classes and *M_ik_*, *M_jk_* are the elements of the disease classes versus biomarker clusters matrices. There are several different methods of hierarchical clustering such as Ward’s method, single, median, complete, average and centroid linkage methods depending on how the distance between clusters is measured. We examined all those methods and got almost the same results. Finally, Ward’s hierarchical clustering is applied (42) because it is considered a better approach (43, 44) and was applied in many other studies.

**Figure 8.**
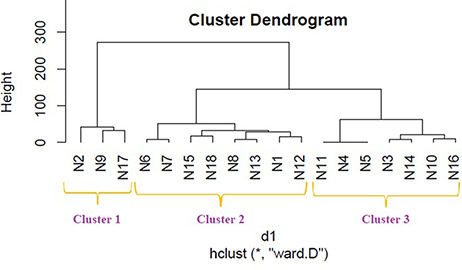
Disease classification dendrogram based on protein biomarkers.

**Figure 9.**
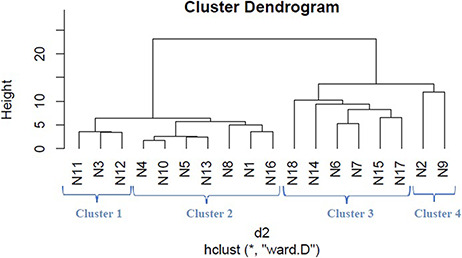
Disease classification dendrogram based on metabolite biomarkers.

We applied hierarchical clustering to Tables 5 and 6 which are prepared, respectively, based on protein and metabolite biomarkers. Figures 8 and 9 show the disease classification dendrograms, respectively, based on protein and metabolite biomarkers.

### Comparison between dendrograms

A dendrogram represents a tree diagram and can display relationships among various objects. We have produced two dendrograms corresponding to two types of biomarkers i.e. protein and metabolite biomarkers (Figures 8 and 9). We compared the similarity between the dendrograms using Baker’s gamma correlation coefficient. We observed the highest similarity corresponding to threshold height 3.

Baker’s gamma coefficient (*Bk*) is the calculation of the Mallows–Fowlkes index for a series of *k* cuts for global comparison of two dendrogram trees (45, 46). A higher value for the Mallows–Fowlkes index means a greater similarity between the benchmark classifications and the clusters. Baker’s gamma coefficient (*Bk*) is an external evaluation method to determine the similarity between two hierarchical clustering’s or a benchmark classification or a clustering. *k* is the desired integer number of cluster groups. To compare our produced dendrograms, we used Baker’s gamma correlation coefficient calculated by ‘dendextend version 1.3.0’ package in the R Language (https://cran.r-project.org/web/packages/dendextend/vignettes/dendextend.html) (46). In this work, we obtained the best coefficient for *k* = 3. ‘*Bk*(*hc*1, *hc*2, *k* = 3)’ function is executed to measure the similarity between two produced dendrograms and the resulting coefficient is 0.4971546 which indicates a very high similarity between two trees. From this high similarity, it can be concluded that in the context of biomarkers, for most diseases the inter disease relations are similar both at the protein level and at the metabolite level. This finding is helpful for understanding the molecular mechanisms of the diseases and narrowing down potential drug candidates for a disease.

#### Relationship among the 18 disease classes

**Figure 10.**
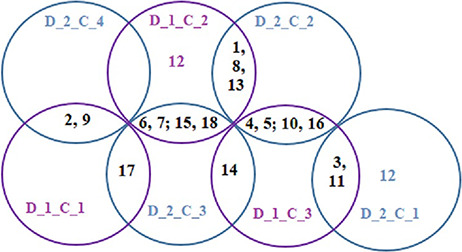
Venn diagrams showing common disease classes between protein and metabolite biomarker-based clusters; 3 magenta circles are the Clusters 1, 2 and 3 of Figure 8 and 4 green circles are the Clusters 1, 2, 3 and 4 of Figure 9.

The Baker’s gamma correlation coefficient value 0.4971546 implies that there is a high similarity between the dendrograms of Figures 8 and 9. We empirically selected 3 and 4 clusters in the dendrograms of Figures 8 and 9, respectively, giving priority to the branching of the dendrogram trees. Figure 10 is drawn based on Figures 8 and 9 showing the common diseases between clusters. In Figure 10, three magenta circles are the Clusters 1, 2, 3 of Figure 8 and four green circles are the Clusters 1, 2, 3, 4 of Figure 9. The disease class IDs that are common between protein and metabolite biomarker based clusters are shown in Figure 10. The disease classes included in any cluster of Figure 8 can be considered to have a similar mechanism at the protein level and the disease classes included in any cluster of Figure 9 can be considered to have similar mechanism at the metabolite level. Considering the common disease classes between the two sets of clusters (Figure 10) and further examining the nearness of the diseases in the dendrograms (Figures 8 and 9), we finally summarize the closely related disease classes as shown in Table 7.

**Table 7.** Groups of closely related disease classes

Group ID	Disease classes ID	Name of disease classes
1	N1	Blood and lymph diseases
	N8	The heart and blood vessels
	N13	The nervous system
2	N2	Cancer
	N9	Diseases of the immune system
3	N3	The digestive system
	N11	Muscle and bone
4	N4	Ear, nose and throat
	N5	Diseases of the eye
5	N6	Female-specific diseases
	N7	Glands and hormones
6	N10	Male-specific diseases
	N16	Skin and connective tissue
7	N15	Respiratory diseases
	N18	Mental and behavioral disorders

N12, N14 and N17 are not included in Table 7, meaning that they are not similar to any other disease class at both the protein and metabolite level according to our study.

Therefore, in the context of biomarkers, it can be concluded that a few diseases belong to different groups at the protein level compared to their cohesion at the metabolite level. However, most disease classes that are similar at the protein level are also similar at the metabolite level. We have surveyed published medical literature to verify evidence to support our findings which are discussed below in terms of the seven groups.

##### Group 1.

Anemia is often connected to heart disease because the heart must pump more blood to make up oxygen through the body, which can cause an enlarged heart or heart failure, high blood pressure and weakening of the heart muscle, rapid or irregular heartbeat (arrhythmia) (47, 48). B-cell chronic lymphocytic leukemia (B-CLL) forms cancer in blood cells, and Ataxia telangiectasia enlarges blood vessels and affects the brain. Approximately 10–20% of B-CLL occurs by ataxia telangiectasia mutated gene damage (49). A low plasma high-density lipoprotein cholesterol (HDL-c) levels in type I Gaucher disease (GD) creates a deficiency of the lysosomal enzyme acid and affects the blood clotting cells (50). HDL-c is also an important risk factor of atherosclerotic disease because blood vessels cannot carry oxygen-rich blood to the heart (51). Cardiovascular QT syndrome disease drug donepezil is also used for Alzheimer disease (AD) patients (52, 53). To predict AD risk, β-amyloid protein 42 and β-amyloid protein 40 in the blood are used (54). Amyotrophic lateral sclerosis (ALS) is a loss of upper and lower motor neurons that affect nerve cells in the brain and spinal cord. Low levels of white blood cell are called CD4 positive T-lymphocytopenia CD4+ T. CD4+ T cells play a neuroprotective role in ALS patients (55, 56). Epilepsy is a disorder which causes seizures due to electrical functioning of the brain. High blood sugar (hyperglycemia) and low blood sugar (hypoglycemia) can affect the nerve cells and low blood glucose can result in a seizure (57, 58). The nervous system is composed of the brain, spinal cord, nerves and ganglia (59). The brain cannot work efficiently without sufficient oxygen and the blood is the carrier of oxygen in the brain (60). Anemia, GD, B-CLL belong to ‘blood and lymph diseases’; enlarged heart or heart failure, high blood pressure and weakening of the heart muscle, rapid or irregular heartbeat (arrhythmia), atherosclerotic disease, QT syndrome belong to ‘the heart and blood vessels’; and AD, ALS, Epilepsy belong to ‘the nervous system’ disease classes. It is noteworthy that Group 1 in Table 7 includes these three disease classes, ‘blood and lymph diseases,’ ‘the heart and blood vessels’ and ‘the nervous system’.

##### Group 2.

Diabetes weakens the patient’s immune system defenses (61). Patients with diabetes risk of developing cancer because insulin is not properly carrying glucose into cells so the pancreas produces more insulin to control blood glucose levels, as a result, the hormone stimulates cell growth (62). Diabetes interrupts DNA and makes the genome unstable that can lead to cancer (63). Diabetes patients have a higher risk of gastric cancer due to a higher reinfection rate of *Helicobacter pylori* (64). Rheumatoid arthritis (RA) is an autoimmune disease that affects joints. RA patients have an excess risk of lung-cancer because of immune function (65, 66). Autoimmune poly glandular syndromes (APS) is a genetic autoimmune disease that has disorders of several endocrine glands and immune-cell dysfunction. APS is associated with thyroid cancer and multi-centric papillary carcinoma (67). Cancer, gastric cancer, and thyroid cancer belong to ‘cancer’ and RA, APS belong to ‘diseases of the immune system’, disease classes. Notice that Group 2 of Table 7 contains these two disease classes, ‘cancer’ and ‘diseases of the immune system’.

##### Group 3.

Inflammatory bowel disease (IBD) is a chronic inflammation of the digestive tract that causes long-lasting ulcers in the intestine (68). IBD is linked with bone density and alterations in bone geometry which is called metabolic bone disease (MBD) (69). Intestinal inflammation and autoimmune associated bone disease are closely connected with hyperactivation of autoreactive CD4 T cells (70). Cystic fibrosis (CF) is a chronic disease that affects the lungs and digestive system. The body produces mucus that obstructs the pancreas. CF-related bone disease (CFBD) is a common complication of CF patients (71). CF patients often have low bone mineral density (BMD) that causes fractures (72). Vitamin D plays a vital role in both CF and BMD (73). Duchenne muscular dystrophy (DMD) is a muscle disorder disease. Gastrointestinal tract (GI) consists of a long tube from our mouth to anus. GI motor function is connected with DMD Patients (74). Myotonic dystrophy (MD) is progressive muscular weakness and affects many other body functions including the GI system, heart and lungs (75). IBD, CF, and the GI belong to ‘the digestive system,’ while MBD, and BMD, DMD, MD belong to the ‘muscle and bone’ disease classes. Therefore, these articles support Group 3 of Table 7, including ‘the digestive system’ and ‘muscle and bone’ disease classes.

##### Group 4.

Cogan’s syndrome is a rheumatic disorder that most commonly affects the eye and the inner ear. Cogan’s syndrome can lead to hearing loss, pain in the eyes, decreased vision, inflammation and vertigo (76). The vestibular (inner ear) and eye movements that act to stabilize gaze are intimately connected through the vestibulo-ocular reflex. Sometimes ear infections with viral or bacterial conjunctivitis can spread to the eyes (77). The eye and nose are linked by the nasolacrimal apparatus and this nasolacrimal apparatus carries tears from the ocular surface to the nose. In many cases, nose disease can affect the eyes and vice versa. For example, allergic rhinitis is an inflammation of the nose which shows watery eyes’ sign (78). Nasal vestibulitis, ‘sinus and nasal polyps’ diseases may cause eye pains because of the tissue around the eye. Moreover, the eyes, nose and cheekbones have the same drains (79). Oculopharyngeal muscular dystrophy is a muscle disorder that slowly affects the upper eyelids and the throat (80). Trachoma is a bacterial infection spread via eye, nose or throat fluids (81). The mentioned diseases mostly viral, bacterial and drainage pathway-related diseases, are associated with each other based on published medical articles. These diseases belong to ‘ear, nose, and throat’ and ‘diseases of the eye’ disease classes. It is worth mentioning that these two disease classes are included in Group 4 of Table 7.

##### Group 5.

Ovarian cancer begins in the ovaries that are obstructed between estrogen and progesterone hormonal balance and create problems in sexual and reproductive development in women (82, 83). Rett syndrome (RTT) is a genetic brain disorder that occurs primarily in girls within 6–18 months of age and causes a disability of language, coordination and repetitive movements. Children with RTT directly interfere with thyroid hormones level (84). Polycystic ovary syndrome is a hormonal disorder which is associated with irregular menstrual cycles, excess facial boils and acne (85). Congenital adrenal hyperplasia is a common genetic disorder of steroidogenesis that affects fertility due to steroid 21-hydroxylase (21 OH) deficiency. Steroid hormones play a significant role in reproductive function and sexual development (86). Hyperthyroidism (overactive thyroid) occurs due to excessive production of the hormone thyroxine by the thyroid gland that causes weight loss and irregular or rapid heartbeat. Graves’ disease causes hyperthyroidism. Thyroid disease occurs often in women than in men (87). Maternal hyperthyroidism increases the risk of miscarriage, premature birth, and a low birth weight baby (88). Uterine fibroids are noncancerous growths of the uterus and endometriosis is cells outside the uterus (89). Both are a common cause of hormone imbalance (90). The above discussions imply that ‘female-specific diseases’ are directly or indirectly related to hormones and responsible for hormonal imbalance. Therefore, ‘glands and hormones’-related diseases are more common for women compared to men. Moreover, women are emotional than men because of hormone fluctuations (91). Interestingly, Group 5 of Table 7 reflects such associations between the ‘female specific’ and ‘glands and hormones’ diseases classes.

##### Group 6.

Male pattern baldness (MPB) is hair loss on the scalp, which is the most common cause of hair loss in men (92). Genes and male sex hormones are mostly responsible for MPB. Moreover, dandruff, scalp skin dryness and skin diseases like psoriasis, allergies and alopecia areata are causes of hair loss (93). Peyronie’s disease or penis curvature is a disorder caused by fibrous scar tissue inside the penis. It may cause bent penis, erectile dysfunction and can make sex uncomfortable or impossible (94). Menkes’ disease (MD) is an X-linked recessive disorder caused by mutations in the ATP7A gene (95). Connective tissue and progressive neurodegeneration are responsible for peculiar ‘kinky’ hair. Moreover, copper deficiency in the body, failure to gain weight, growth and nervous system deterioration are the main characteristic of MD. Patients with MD are the vast majority in males more than in females (96). The above-mentioned diseases MPB and, Peyronie’s disease belong to ‘male-specific diseases,’ while MD belong to the ‘skin and connective tissue’ disease class. We found some ‘male-’ and ‘tissue’-related diseases which are linked with female, blood, muscle, nervous system diseases and so on. But more connections are found within male and tissue-related diseases. The above statements about diseases in the ‘male-specific’ and ‘skin and connective tissue’ disease classes are supported by Group 6 of Table 7.

##### Group 7.

Asthma is a chronic disease of the respiratory system in which airways swell or narrow or produce extra mucus that causes breathing difficulties. Bipolar disorder is a mental disorder that includes lows of depression, mania or hypomania (feeling high) and unusual shifts in mood. Severe asthma is associated with bipolar disorder, anxiety disorders, post-traumatic stress and severe mental disorder. Asthma and bipolar disorder share a similar pathophysiology, and a patient with asthma has 2.12 times higher risk of bipolar disorder (97). Alpha-1 antitrypsin deficiency (A1AD) is a genetic disorder that causes lung and liver disease. Anxiety disorders are a group of mental disorders including anxiety, fear, panic, specific phobias, agoraphobia, worry about future events and social anxiety disorder. Emotional and anxiety disorders are common comorbidities in alpha-1 antitrypsin deficiency (AATD) patients (98). Schizophrenia is a brain disorder that can cause delusions, hallucinations and extremely disordered thinking and affects how a person feels, thinks and behaves. Chronic obstructive pulmonary disease (COPD) is a group of lung diseases that causes breathing difficulties and poor airflow. Schizophrenia is connected with weakened lung function and increases the risk of COPD and pneumonia (99). Marijuana (Cannabis) and tobacco smoke pollute the lungs and reduce brain activity and the volume of brain regions. Marijuana addicted people are attacked by both respiratory and mental disorders (100). Asthma, A1AD and COPD belong to ‘respiratory diseases,’ while bipolar disorder and anxiety disorders, and schizophrenia belong to the ‘mental and behavioral disorders’ disease classes. Diseases in both these disease classes are very close to each other according to medical research, and our study also grouped them in Group 7 of Table 7.

## Conclusions

In the present study, we have developed a human biomarker database, which can be accessed online at the KNApSAcK family database site (http://www.knapsackfamily.com/Biomarker/top.php). Our team collected and verified data from reliable articles. All of the biomarker information sources are linked to valid references. The database may be useful for the research on proteins, metabolites, disease patterns, diseases similarities, novel drug discovery and drug characteristic research, and it will play a vital role in personalized medicine (PM). Moreover, within a short time, without doing a literature survey, a researcher can get biomarker information from a single platform, instead of searching multiple sources. In the developed database, there are 1686 protein and 495 metabolite biomarkers involving, respectively, 3693 and 846 diseases–biomarker associations. Apart from the database development, we have examined disease–disease relations in an upper hierarchy, i.e. at the NCBI disease class level. Disease–disease relations provide clues to understanding disease mechanisms, drug design, etc., because similar diseases share similar pathways and genes. We have adopted two approaches based on protein and metabolite biomarkers to classify the diseases and found a remarkable consistency between the results we obtained. Baker’s gamma correlation value of 0.4971546 was obtained between the dendrograms generated by the two approaches. We have collected FASTA files of protein biomarkers and SDF files of metabolite biomarkers, then extracted descriptors and fingerprints using the programming language R. We have used the PCC and Tanimoto coefficient to calculate the similarities between protein and metabolite biomarkers, respectively. The network clustering algorithm DPClusO and hierarchical clustering were applied to extract associations among 18 disease classes. Finally, we determined seven groups involving 15 of the 18 disease classes based on disease similarities in both protein and metabolite levels. We thoroughly studied medical literature and gathered substantial evidence to support our findings. To our knowledge, this is one of the first approaches to classify diseases based on biomarkers. Our results are useful to find and explain inter-disease interactions, disease pathways and novel drugs.

## Abbreviations

*PM*, precision medicine; *NIH*, National Institutes of Health; *NCBI*, The National Center for Biotechnology Information; *PCC*, Pearson correlation coefficient; *Bk*, Baker’s gamma coefficient; *B-CLL*, B-cell chronic lymphocytic leukemia; *AT*, Ataxia telangiectasia; *HDL-c*, high-density lipoprotein cholesterol; *GD*, Gaucher disease; *AD*, Alzheimer Disease; *H. pylori*, Helicobacter pylori; *RA*, Rheumatoid arthritis; *APS*, Autoimmune poly glandular syndromes; *IBD*, Inflammatory bowel disease; *CF*, Cystic fibrosis; *CFBD*, Cystic fibrosis-related bone disease; *BMD*, bone mineral density; *DMD*, Duchenne muscular dystrophy; *GI*, Gastrointestinal tract; MD, Myotonic dystrophy; *VOR*, Vestibulo-ocular reflex; *RTT*, Rett syndrome; *TH*, thyroid hormones; *MPB*, Male pattern baldness; *MD*, Menkes disease; *A1AD*, Alpha-1 antitrypsin deficiency; *COPD*, Chronic obstructive pulmonary disease.
